# Intra-abdominal Desmoid Tumors Mimicking Gastrointestinal Stromal Tumor (GIST) Recurrence: A Case Report

**DOI:** 10.7759/cureus.100294

**Published:** 2025-12-28

**Authors:** Yoko Senaha, Takeshi Matsubara, Shunsuke Kaji, Hikota Hayashi, Masaaki Hidaka

**Affiliations:** 1 Department of Digestive and General Surgery, Shimane University, Izumo, JPN

**Keywords:** desmoid tumor, gastrointestinal stromal tumor (gist), histopathological analysis, imaging modalities, intra-abdominal mass

## Abstract

Desmoid tumors, also known as aggressive fibromatosis, are rare, benign soft tissue neoplasms characterized by local invasiveness and a high recurrence rate. They can mimic malignant tumors, particularly in patients with a history of gastrointestinal stromal tumors (GISTs).

We report a case of a 60-year-old male who developed intra-abdominal desmoid tumors five years after undergoing laparoscopy and endoscopy cooperative surgery (LECS) for gastric GIST, followed by adjuvant imatinib therapy. Imaging studies, including contrast-enhanced computed tomography (CT) and positron emission tomography-computed tomography (PET-CT), revealed two mesenteric masses. The larger lesion demonstrated elevated fluorodeoxyglucose (FDG) uptake (SUVmax = 6.7), raising suspicion for recurrent GIST. Surgical resection was performed, and histopathological examination confirmed the diagnosis of desmoid tumors. Immunohistochemical analysis showed positive β-catenin staining, with negative markers for CD34, desmin, and c-kit, distinguishing it from recurrent GIST.

This case highlights the diagnostic challenge of differentiating desmoid tumors from GIST recurrence based on imaging alone. Histopathological confirmation remains crucial for accurate diagnosis. Surgical resection is the primary treatment for symptomatic desmoid tumors, but given their high recurrence rate, long-term follow-up and a multidisciplinary approach are essential for optimal management.

## Introduction

Desmoid tumors, also referred to as aggressive fibromatosis, are rare, benign soft tissue neoplasms that originate from fascial or musculoaponeurotic structures. Although they lack metastatic potential, they demonstrate locally aggressive behavior and have a high recurrence rate of 20%-68% following surgical resection. Desmoid tumors account for approximately 0.03% of all neoplasms and about 3% of all soft tissue tumors [1,2]. They may arise sporadically or in association with genetic conditions such as familial adenomatous polyposis (FAP), particularly Gardner syndrome. These tumors can occur in various anatomical sites, including the abdominal wall, extra-abdominal locations, and, less commonly, the intra-abdominal region [3,4].

Intra-abdominal desmoid tumors are uncommon, representing only about 8% of all desmoid tumors [3]. They frequently occur in patients with FAP but may also arise sporadically, often following abdominal surgery, trauma, or pregnancy [3]. The association with previous surgical procedures is clinically significant, as desmoid tumors may mimic malignant lesions, particularly in patients with a history of cancer. Therefore, accurate diagnosis typically requires a combination of imaging, histopathological and, in selected cases, molecular analyses to distinguish desmoid tumors from malignancies such as recurrent gastrointestinal stromal tumors (GISTs).

GIST is the most common mesenchymal tumor of the gastrointestinal tract, and patients with high-risk GIST require long-term surveillance due to the potential for recurrence or metastasis [5]. The modified Fletcher/Joensuu classification [6] provides a framework for risk stratification; however, recurrence may still occur even after curative resection, particularly in high-risk cases. Because desmoid tumors can closely resemble GIST recurrence both clinically and radiologically, differentiation between these entities remains a major diagnostic challenge.

In this report, we present a rare case of intra-abdominal desmoid tumors that developed five years after laparoscopy and endoscopy cooperative surgery (LECS) for gastric GIST. This case illustrates the diagnostic difficulty of distinguishing desmoid tumors from GIST recurrence and underscores the importance of including desmoid tumors in the differential diagnosis of newly detected intra-abdominal masses in patients with a history of GIST.

## Case presentation

**Figure 1:**
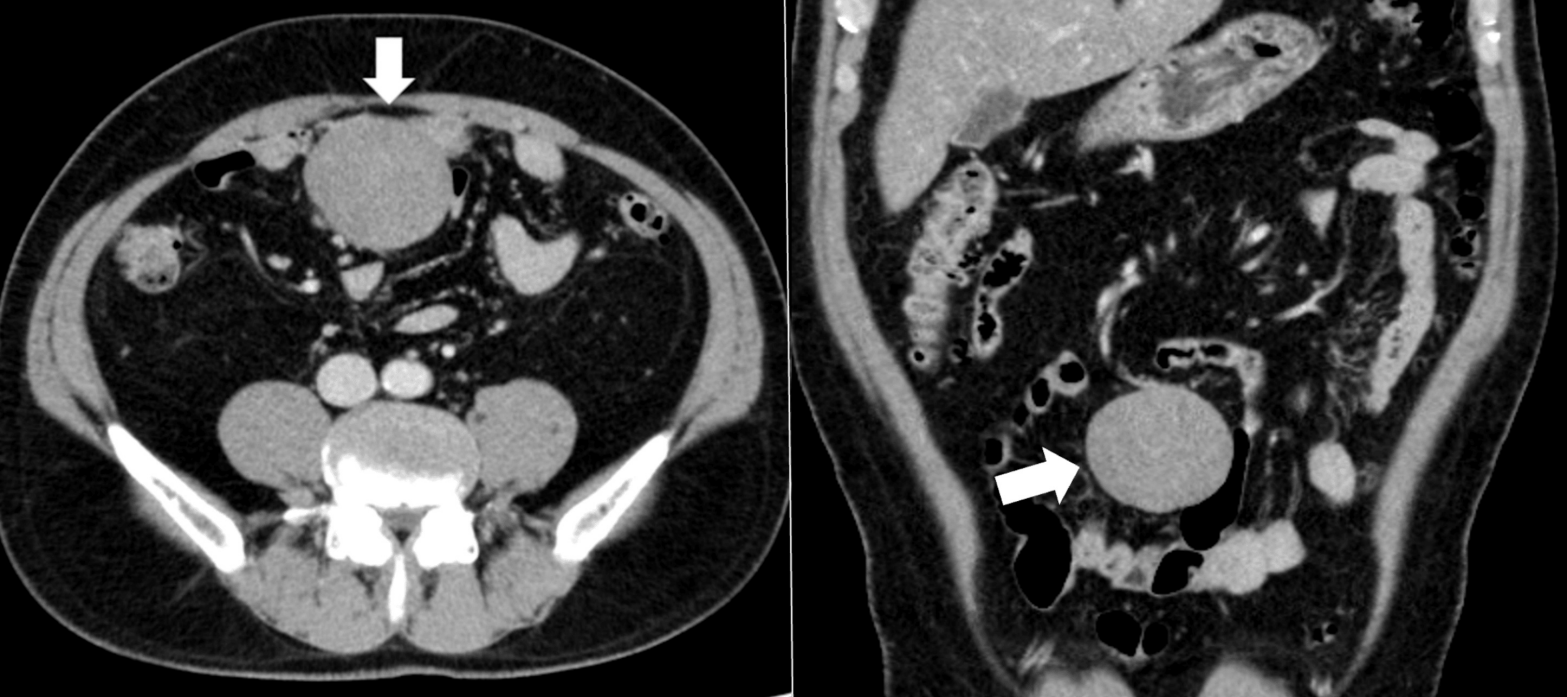
Contrast-enhanced CT scan of lesion 1 showing heterogeneous internal enhancement, measuring 6 cm in diameter (white arrows). CT, computed tomography

**Figure 2:**
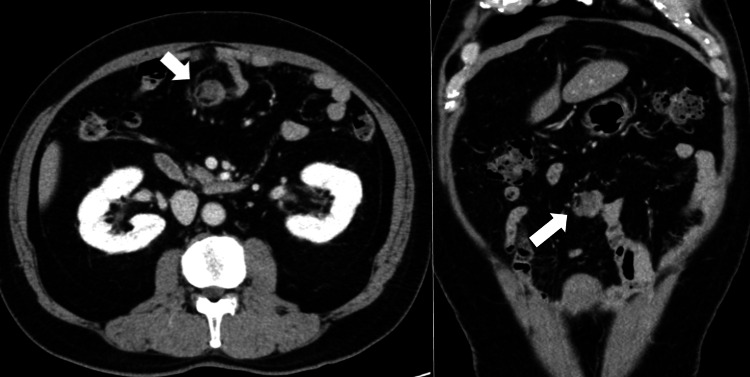
Contrast-enhanced CT scan of lesion 2, measuring 2 cm in diameter (white arrows). CT, computed tomography

**Figure 3:**
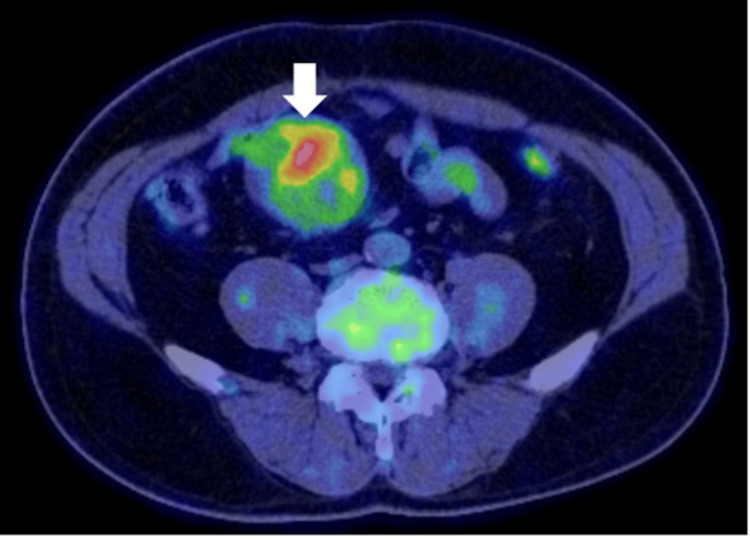
PET-CT scan of lesion 1 showing increased fluorodeoxyglucose uptake in the lesion of tumors (white arrow). PET-CT, positron emission tomography-computed tomography

The patient is a 60-year-old male with a history of gastric GIST, diagnosed five years prior. LECS was performed, followed by three years of adjuvant imatinib therapy. The tumor was classified as high-risk according to the modified Fletcher/Joensuu classification; its size was 2.3 × 1.9 cm, and the mitotic count was >10/50 high-power fields. During the five-year follow-up, contrast-enhanced computed tomography (CT) and positron emission tomography-computed tomography (PET-CT) scans were used to evaluate the newly discovered masses (Figures 1-3).

While CT showed two solid tumors in the mesentery, measuring 60 mm and 20 mm, PET-CT revealed increased fluorodeoxyglucose (FDG) uptake in the larger mass (SUVmax = 6.7). The high SUV value raised concerns about malignancy, reinforcing the suspicion of recurrent GIST, as malignant tumors typically show increased metabolic activity. Gadolinium-enhanced magnetic resonance imaging (MRI) in this case revealed heterogeneous signal patterns, with hypointense centers and hyperintense peripheries on T2-weighted images.

While such findings could suggest a recurrent GIST, they are also consistent with desmoid tumors, underscoring the diagnostic complexity. 

**Figure 4:**
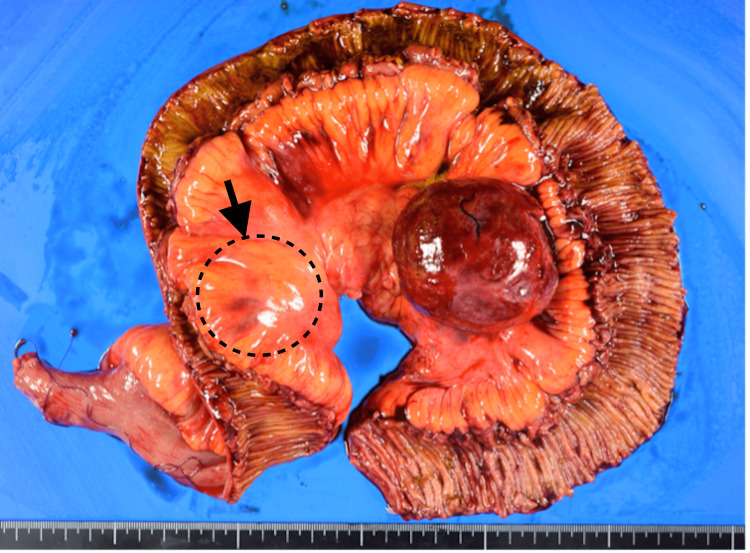
The specimen of resection, where the area enclosed by a dotted line contains another small mass, is shown.

Given the imaging findings, surgical resection was deemed necessary to remove the suspected recurrent tumors and provide histological clarity. Intraoperatively, two firm, well-circumscribed masses were found within the mesentery. The larger mass was adherent to the marginal artery and mesenteric vein, necessitating the resection of 110 cm of the jejunum (Figure 4).

Histopathological analysis of the resected masses revealed the characteristic features of desmoid tumors. On hematoxylin and eosin staining, the tumors consisted of spindle-shaped cells arranged within an eosinophilic collagenous stroma. No significant nuclear atypia or mitotic activity was observed, confirming the benign nature of the tumors.

**Figure 5:**
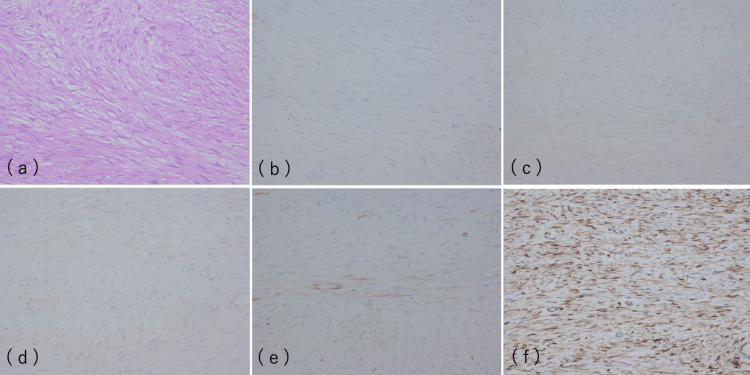
a) HE stain ×200: the tumors consisted of spindle-shaped cells arranged within an eosinophilic collagenous stroma; b) CD34 stain ×200: negative; c) Desmin stain ×200: negative; d) c-kit stain ×200: negative; e) α-SMA stain ×200: focal positivity for α-SMA; f) β-catenin stain ×200: positive results for β-catenin. HE, hematoxylin and eosin; α-SMA, α-smooth muscle actin

Immunohistochemical staining was performed to further differentiate the masses from GIST recurrence. The tumors showed strong nuclear positivity for β-catenin, a key marker of desmoid tumors (Figure 5), while being negative for CD34, desmin, and c-kit (CD117), markers typically associated with GIST. Additionally, focal positivity for α-smooth muscle actin (α-SMA), a marker of smooth muscle differentiation, was noted.

These findings conclusively ruled out the possibility of GIST recurrence and confirmed the diagnosis of intra-abdominal desmoid tumors. 

## Discussion

Desmoid tumors, despite their benign histology, present significant clinical challenges due to their local invasiveness and propensity to recur [1,7]. Intra-abdominal desmoid tumors, though rare, can mimic recurrent malignancies, especially in patients with a history of neoplasms such as GIST. The case presented here illustrates the diagnostic difficulties in differentiating desmoid tumors from GIST recurrence, based on imaging alone. Both tumor types can present with overlapping features on CT, MRI, and PET-CT, making histopathological examination essential for accurate diagnosis.

Imaging modalities such as contrast-enhanced CT and PET-CT, while helpful in identifying the presence of tumors, are limited in their ability to distinguish between benign and malignant lesions definitively. In this case, increased FDG uptake on PET-CT (SUVmax = 6.7) highly suggests malignancy, often seen in recurrent GIST. However, desmoid tumors can also show FDG uptake, albeit to a lesser extent than malignant neoplasms, which can lead to misinterpretation of results. MRI, with its ability to provide detailed soft tissue characterization, further demonstrated the variable imaging features of desmoid tumors, but no single imaging modality can reliably differentiate these tumors from malignancies. Kim et al. reported that their 35 desmoid cases of postoperative GIST occurred near the previous surgical site [8]. However, as in our case, desmoid tumors may occasionally arise at a site distant from the original surgical location, which is uncommon [2]. In such cases, desmoid tumors imitate postoperative recurrence, such as lymph node metastasis or peritoneal dissemination.

Histopathological analysis, supported by immunohistochemical staining, remains the gold standard for diagnosing desmoid tumors. The positive β-catenin staining observed in this case is a hallmark of desmoid tumors, particularly in those with mutations in the CTNNB1 gene [3,6,7]. The absence of CD34, c-kit, and desmin, markers commonly associated with GIST, helped distinguish the desmoid tumors from recurrent GIST. This case highlights the importance of integrating histopathology with imaging findings in the postoperative surveillance of GIST patients to avoid misdiagnosis.

The management of desmoid tumors is challenging due to their unpredictable behavior. Surgical resection with negative margins is the preferred treatment for symptomatic or enlarging desmoid tumors, particularly in cases where the tumors involve critical structures such as the mesentery [8,9]. However, desmoid tumors have a high recurrence rate, even after margin-negative resection, with recurrence rates ranging from 20% to 68%. Given the aggressive nature of desmoid tumors and the complexity of resection in this case (requiring 110 cm of jejunal resection) [10-12], careful long-term follow-up is warranted. Regular imaging, typically with CT or MRI, is recommended every six months post-surgery for the first few years, to monitor for recurrence.

In cases where surgery is not feasible, or in patients with recurrent disease, alternative management strategies, such as active surveillance, systemic therapy, or radiation therapy, may be considered. Recent studies have suggested that a "wait-and-see" approach can be practical for asymptomatic or stable desmoid tumors, which may remain unchanged or even regress spontaneously over time [13,14]. Systemic therapies, including nonsteroidal anti-inflammatory drugs (NSAIDs), hormonal agents (such as antiestrogens), and tyrosine kinase inhibitors (e.g., imatinib), have shown promise in managing unresectable or recurrent desmoid tumors [14]. However, more research is needed to establish standardized treatment protocols and to identify reliable prognostic factors for recurrence.

## Conclusions

In conclusion, this case emphasizes the diagnostic and therapeutic challenges associated with intra-abdominal desmoid tumors, particularly in patients with a history of GIST or prior abdominal surgery. While imaging is crucial in detecting new tumors, definitive diagnosis often requires histopathological and immunohistochemical confirmation. Surgical resection remains the cornerstone of treatment for desmoid tumors, but, given the high recurrence rates, a multidisciplinary approach involving active surveillance and systemic therapy should be considered in selected cases. Further research is needed to clarify the emerging therapies' role and improve prognostic models for predicting recurrence and guiding management decisions.
